# A Practical Scheme

**Published:** 1887-06

**Authors:** 


### A PRACTICAL SCHEME.

At the meeting of the Illinois State Dental Society for 1886, Dr. G. V. Black, of Jacksonville, at the invitation of the society, presented his apparatus for the study of microbes, and made cultures before the society, detailing his methods and the results of his observations. It was then remarked that it seemed a pity that one who exhibited such an enthusiastic love for experimental investigation, such an aptitude for scientific research, and who was so thoroughly qualified by previous training for such work, should not be able to devote his whole time to it. It was said that there were plenty who could earn money, but there were few who, like

Miller and Black, were fitted for profitable scientific research. Such men, if they were relieved of everything else, could do more for science than all the desultory efforts of those who were comparatively untrained could accomplish in double the time. It would be more profitable, both for the profession at large and for the individual members, to place the money which each was prepared to spend in research in one common fund, and at the disposal of experts who would give to others the benefits of their studies, than it would be to fritter it away in inconsecutive and too often unscientific experiments.

The remarks were well received, and have borne fruit in the appointment of a committee, consisting of Drs. T. W. Brophy, Edgar D. Swain, C. A. Kitchen, J. Frank Marriner, Geo. H. Cushing, A. W. Harlan and T. L. Gilmer, who are charged with the duty of raising a sum of money to be devoted to original scientific investigations, and to be expended under the direction of the committee. A considerable sum has already been received, and we believe it is the purpose of the committee to place it in the hands of Dr. Black, with the request that he expend it in such way as shall best further the interests of scientific dental practice. It is suggested that he visit Europe, where he will have advantages for research such as this country does not afford.

We have not learned whether Dr. Black will temporarily give up his practice for this purpose, but cannot conceive that he will decline an offer so honorable, and a work which must be so congenial to his tastes, and for which he is so well fitted. The proposition is not only a flattering one for Dr. Black, but it is greatly to the honor of the western dentists who are engaged in furthering it. Such a scheme must commend itself to every progressive dentist, and it cannot but be that there are many who will desire a part in its accomplishment. If any wish to assist in so honorable and practical a work, they have but to address Dr. Brophy at Chicago.

In the capital report of the meeting of the Illinois State Dental Society for 1887, the first part of which is published in this number, will be found an account of the work and observations of Dr. Black during the past year, as reported to that society from day to day during the sessions. We need not commend it to our readers, for like good wine it needs no bush, and will be sure to attract their attention.

